# Socioeconomic Outcome and Quality of Life in Adults after Status Epilepticus: A Multicenter, Longitudinal, Matched Case–Control Analysis from Germany

**DOI:** 10.3389/fneur.2017.00507

**Published:** 2017-09-26

**Authors:** Lena-Marie Kortland, Susanne Knake, Felix von Podewils, Felix Rosenow, Adam Strzelczyk

**Affiliations:** ^1^Epilepsy Center Hessen, Philipps-University, Marburg, Germany; ^2^Epilepsy Center Greifswald, Ernst-Moritz-Arndt-University, Greifswald, Germany; ^3^Epilepsy Center Frankfurt Rhine-Main, Goethe-University, Frankfurt am Main, Germany

**Keywords:** epilepsy, seizure, anticonvulsants, morbidity, mortality

## Abstract

**Background:**

There is a lack of data concerning socioeconomic outcome and quality of life (QoL) in patients after status epilepticus (SE) in Germany.

**Patients and methods:**

Adult patients treated between 2011 and 2015 due to SE at the university hospitals in Frankfurt, Greifswald, and Marburg were asked to fill out a questionnaire regarding long-term outcome of at least 3 months after discharge. The SE cohort consisted of 25.9% patients with an acute symptomatic, 42% with a remote symptomatic and previous epilepsy, 22.2% with a new-onset remote symptomatic, and 9.9% with other or unknown etiology. A matched case–control analysis was applied for comparison with patients with drug refractory epilepsy and seizure remission, both not previously affected by SE.

**Results:**

A total of 81 patients (mean age: 58.7 ± 18.0 years; 58% female) participated. A non-refractory course was present in 59.3%, while 27.2% had a refractory SE (RSE) and 13.6% had a superrefractory SE (SRSE). Before admission, a favorable modified Rankin Scale (mRS) of 0–3 was found in 82.7% (67/81), deteriorating to 38.3% (31/81) (*p* = 0.003) at discharge. The majority returned home [51.9% (42/81)], 32.1% entered a rehabilitation facility, while 12.3% were transferred to a nursing home and 3.7% to another hospital. The overall mRS at follow-up did not change; 61.8% (45/74) reached an mRS of 0–3. In RSE and SRSE, the proportion with a favorable mRS increased from 45.5% at discharge to 70% at follow-up, while QoL was comparable to a non-refractory SE course. Matched epilepsy controls in seizure remission were treated with a lower mean number of anticonvulsants (1.3 ± 0.7) compared to controls with drug refractory epilepsy (1.9 ± 0.8; *p* < 0.001) or SE (1.9 ± 1.1; *p* < 0.001). A major depression was found in 32.8% of patients with SE and in 36.8% of drug refractory epilepsy, but only in 20.3% of patients in seizure remission. QoL was reduced in all categories (QOLIE-31) in SE patients in comparison with patients in seizure remission, but was comparable to patients with drug refractory epilepsy.

**Discussion:**

Patients after SE show substantial impairments in their QoL and daily life activities. However, in the long term, patients with RSE and SRSE had a relatively favorable outcome comparable to that of patients with a non-refractory SE course. This underlines the need for efficient therapeutic options in SE.

## Introduction

Status epilepticus (SE) presents as a major neurological emergency and is associated with a substantial burden on individuals and society (1–3). Prolonged inpatient treatment and neurological sequelae due to SE lead to substantial direct and indirect costs and result in reduced quality of life (QoL). Rehabilitation and informal care are often necessary following discharge from acute treatment and might result in further costs. Studies on outcome show a substantial portion of patients who are discharged with a neurologic deficit, while overall hospital mortality is about 15–20%. Both SE related morbidity and mortality increase with a refractory course and prolonged inpatient treatment (4–6). Prehospital and in-hospital treatment strategies aim at a timely cessation of seizure activity and consist of benzodiazepines, intravenous anticonvulsants, and anesthetic drugs in selected cases (7).

Given the reduced QoL and increased rate of depression in patients with drug refractory epilepsy, as proven by several studies (8–11), patients with SE are very likely to suffer from decreased QoL and mood disorders. However, there is a paucity of data concerning socioeconomic outcome and QoL in patients after an SE, especially as there is no study on this topic from Germany.

Thus, the objective of this multicenter study is to determine the outcome, resource utilization, and QoL indicators following an episode of SE. For comparison, patients suffering from epilepsy who had never previously experienced an episode of SE were matched by age and gender: we matched two groups, one with a drug refractory epilepsy (DRE) and one with epilepsy patients in seizure remission (SRE) for more than 1 year as they show distinct outcomes regarding QoL, health resource utilization, and mood disorders.

## Patients and Methods

### Study Settings and Design

This longitudinal study on outcome, QoL, and resource utilization was performed by means of a bottom-up approach from the perspective of the statutory health insurer at the university hospitals in Frankfurt am Main, Greifswald, and Marburg. The study was granted approval by the local ethics committees and registered at the German Clinical Trials Register (DRKS00008718). The Strengthening the Reporting of Observational Studies in Epidemiology (STROBE) guidelines were followed (12).

Adult patients of 18 years or older treated due to SE at the participating university hospitals during the 5-year study period of 2011–2015 were asked to fill out a questionnaire regarding long-term outcome for at least 3 months after discharge. Overall, 669 patients were treated due to SE during the study period with an average in-hospital mortality of 18.8% (*n* = 126). Therefore, responder rate was 15% regarding 81 of 543 survivors. The questionnaire was validated for use in people with epilepsy (13, 14). Patients with SE received standard care with no intervention due to the study, and a decision for rehabilitation after SE was at the discretion of the treating physician. An operational definition consistent with ILAE guidelines was adopted that defines convulsive SE as ≥5 min of continuous seizure or two or more discrete seizures, between which there is an incomplete recovery (15, 16). In case of focal SE or absence SE, the definition encompassed at least 5 min of seizure duration; however, none of the patients were identified with a focal SE or absence SE below duration of 20 min (1). Refractory SE (RSE) was defined as recurrent seizure activity despite two appropriately selected and dosed antiepileptic drugs, including benzodiazepine, and superrefractory SE (SRSE) was referred to as a SE that continues or recurs 24 h or more after the initiation of treatment with anesthetic drugs, including cases in which seizure control is attained after induction of anesthesia, but recurs on weaning the patient off the anesthetic agent (4, 17, 18). Patients were assigned to four major groups based on etiology and onset of SE with (1) acute symptomatic SE, due to an acute brain injury as defined by the ILAE (19); (2) new-onset, remote symptomatic SE, with no history of epilepsy or SE; (3) remote symptomatic SE, with history of epilepsy or SE; and (4) other etiologies, such as idiopathic generalized epilepsy or progressive symptomatic causes. The epilepsy diagnosis was based on the definitions proposed by the ILAE and the International Bureau for Epilepsy (20). Patients were excluded if the diagnosis of SE could not be unequivocally determined, or if SE was due to hypoxia after cardiopulmonary resuscitation.

We employed a matched case–control analysis to compare the SE group with two control groups of epilepsy patients, either with drug refractory epilepsy (DRE) or in seizure remission (SRE) for more than 1 year. None of the patients from the epilepsy control groups suffered from an SE during their lifetime. Patients were matched by age and gender. The distribution of age and gender did not differ significantly across the groups, except for SRE patients, who were, in mean, 2 years younger than patients in the SE group.

### Instruments

To analyze the health-related QoL, we used scales, such as Quality of Life in Epilepsy Inventory (QOLIE-31) (21), Neurological Disorders Depression Inventory for Epilepsy (NDDI-E) (22), A-B neuropsychological assessment schedule (ABNAS, originally the A-B neurotoxicity scale) (23), Liverpool Adverse Events Profile (LAEP) (24), and the EuroQol questionnaire (EQ-5D) (25). Measures of severity of illness and long-term outcome included the modified Rankin Scale (mRS) (26) on admission, discharge, and follow-up. Healthcare resource utilization is reported as use of inpatient, outpatient, rehabilitation, and anticonvulsive treatment. For cost unit data and details of evaluation, please refer to previous publications (27, 28).

### Data Entry and Statistical Analysis

Statistical analyses were performed using IBM SPSS Statistics, version 22.0 (IBM Corp., Armonk, NY, USA). Most data are presented as percentage or mean ± SD and as minimum and maximum. Comparisons between groups were accomplished using adequate parametric and non-parametric tests. Since the study was planned to have an explorative nature, no further adjustment for multiple testing was performed.

## Results

### Characteristics of Patients with SE

During the 5-year study period, a total of 81 patients (mean age: 58.7 ± 18.0 years, range: 21–97 years; 58.0% female) participated in the study. An acute symptomatic etiology was present in 25.9% (*n* = 21), a remote symptomatic SE was attributed in 22.2% (*n* = 18) with new-onset SE, and in 42.0% (*n* = 34) of patients with history of epilepsy, other or unknown etiologies were seen in 9.9% (n = 8). Of these cases, 59.3% (*n* = 48) had a non-RSE, 27.2% (*n* = 22) an RSE, and 13.6% (*n* = 11) had an SRSE. Details of clinical or socioeconomic characteristics and QoL at least 3 months after SE are provided in Table 1.

**Table 1:** Socioeconomic and clinical characteristics in patients with SE provided for all patients (*n* = 81) and according to a non-RSE (*n* = 48) and RSE or SRSE course (*n* = 33).

	All patients with SE (*n* = 81)	Non-RSE (*n* = 48)	RSE/SRSE (*n* = 33)	*p* Value^a^
Age (years) at admission				
Mean ± SD	58.7 ± 18.0	61.8 ± 16.8	54.2 ± 18.9	0.063
Range	21–97	23–97	21–80	
Sex	% (*n*)	% (*n*)	% (*n*)	
Male	42.0 (34)	35.4 (17)	51.5 (17)	0.225
Female	58.0 (47)	64.6 (31)	48.5 (16)	
Etiology	% (*n*)	% (*n*)	% (*n*)	
Acute symptomatic	25.9 (21)	16.7 (8)	39.4 (13)	0.021
Remote symptomatic	42.0 (34)	43.8 (21)	39.4 (13)	(Acute vs non-acute)
New onset SE	22.2 (18)	27.1 (13)	15.2 (5)	
Other or unknown	9.9 (8)	12.5 (6)	6.1 (2)	
mRS at admission	% (*n*)	% (*n*)	% (*n*)	
mRS 0–3	82.7 (67)	81.3 (39)	84.8 (28)	0.673
mRS 4–5	17.3 (14)	18.8 (9)	15.2 (5)	
mRS at discharge	% (*n*)	% (*n*)	% (*n*)	
mRS 0–3	61.7 (50)	72.9 (35)	45.5 (15)	0.012
mRS 4–5	38.3 (31)	27.1 (13)	54.5 (18)	
mRS at follow-up	% (*n*)	% (*n*)	% (*n*)	
mRS 0–3	60.8 (45)	54.5 (24)	70.0 (21)	0.180
mRS 4–5	39.2 (29)	45.5 (20)	30.0 (9)	
Discharge destination	% (*n*)	% (*n*)	% (*n*)	
Home	51.9 (42)	64.6 (31)	33.3 (11)	0.013
Rehabilitation	32.1 (26)	16.7 (8)	54.5 (18)	(home vs other)
Other hospital	3.7 (3)	2.1 (1)	6.1 (2)	
Nursing home	12.3 (10)	16.7 (8)	6.1 (2)	
Living situation	% (*n*)	% (*n*)	% (*n*)	
At home without help	25.9 (21)	27.1 (13)	24.2 (8)	0.732
At home with help (family, nursing, etc.)	54.0 (43)	47.9 (23)	60.6 (20)	
Nursing home	19.8 (16)	22.9 (11)	15.2 (5)	
n.a.	(1)	(1)		
Care level	% (*n*)	% (*n*)	% (*n*)	
None	33.3 (27)	33.3 (16)	33.3 (11)	0.947
None, but in need of care	8.6 (7)	10.4 (5)	6.1 (2)	
Care level existing	56.8 (46)	54.2 (26)	60.6 (20)	
n.a.	(1)	(1)		
Grade of disability	% (*n*)	% (*n*)	% (*n*)	
Yes	79.0 (64)	79.2 (38)	78.8 (26)	0.820
None	19.8 (16)	18.8 (9)	21.2 (7)	
n.a.	(1)	(1)		
Number of AEDs at discharge	% (*n*)	% (*n*)	% (*n*)	
0	1.2 (1)	-	3.0 (1)	
1	33.3 (27)	43.8 (21)	18.2 (6)	
2	32.1 (26)	35.4 (17)	27.3 (9)	
≥3	33.3 (27)	20.8 (10)	51.5 (17)	
Total (mean ± SD)	2.1 ± 1.0	1.8 ± 0.9	2.5 ± 1.1	0.007
Number of AEDs at follow-up	% (*n*)	% (*n*)	% (*n*)	
0	4.2 (3)	2.4 (1)	6.7 (2)	
1	40.3 (29)	50.0 (21)	26.7 (8)	
2	19.4 (14)	23.8 (10)	13.3 (4)	
≥3	36.1 (26)	23.8 (10)	53.3 (16)	
Total (mean ± SD)	1.9 ± 1.1	1.8 ± 1.0	2.2 ± 1.1	0.113
Healthcare resource utilization within the last 3 months				
Inpatient treatment due to epilepsy	% (*n*)	% (*n*)	% (*n*)	
Yes	22.2 (18)	17.0 (8)	30.3 (10)	0.146
None	77.7 (63)	83.0 (40)	69.7 (23)	
Outpatient hospital treatment due to epilepsy	% (*n*)	% (*n*)	% (*n*)	
Yes	14.3 (11)	11.1 (5)	18.8 (6)	0.316
None	85.7 (70)	88.9 (43)	81.3 (27)	
NDDI-E	% (*n*)	% (*n*)	% (*n*)	
Major depression (Score >15)	32.8 (19)	30.3 (10)	36.0 (9)	0.647
No major depression (Score ≤15)	67.2 (39)	69.7 (23)	64.0 (16)	
ABNAS				
Total (mean ± SD)	34.9 ± 20.8	34.6 ± 20.1	35.2 ± 22.0	0.351
Range	0–72	3–71	0–72	
LAEP				
Total (mean ± SD)	41.9 ± 10.4	41.5 ± 10.3	42.3 ± 10.8	0.997
Range	14–63	20–62	14–63	
QOLIE-31				
Overall T (mean ± SD)	43.5 ± 13.7	44.2 ± 13.9	42.5 ± 13.6	0.870
Range	11–73	11–73	16–66	
VAS (mean ± SD)	48.3 ± 24.5	49.2 ± 22.7	47.2 ± 27.0	0.618

*^a^Comparison between non-RSE and RSE/SRSE*.

*SE, status epilepticus; RSE, refractory SE; SRSE, superrefractory SE; mRS, modified Rankin Scale; AEDs, antiepileptic drugs; NIDDI-E, Neurological Disorders Depression inventory for Epilepsy; LAEP, Liverpool Adverse Events Profile; QOLIE, Quality of Life in Epilepsy Inventory; VAS, visual analog scale; ABNAS, A-B neuropsychological assessment schedule*.

**Figure 1:**
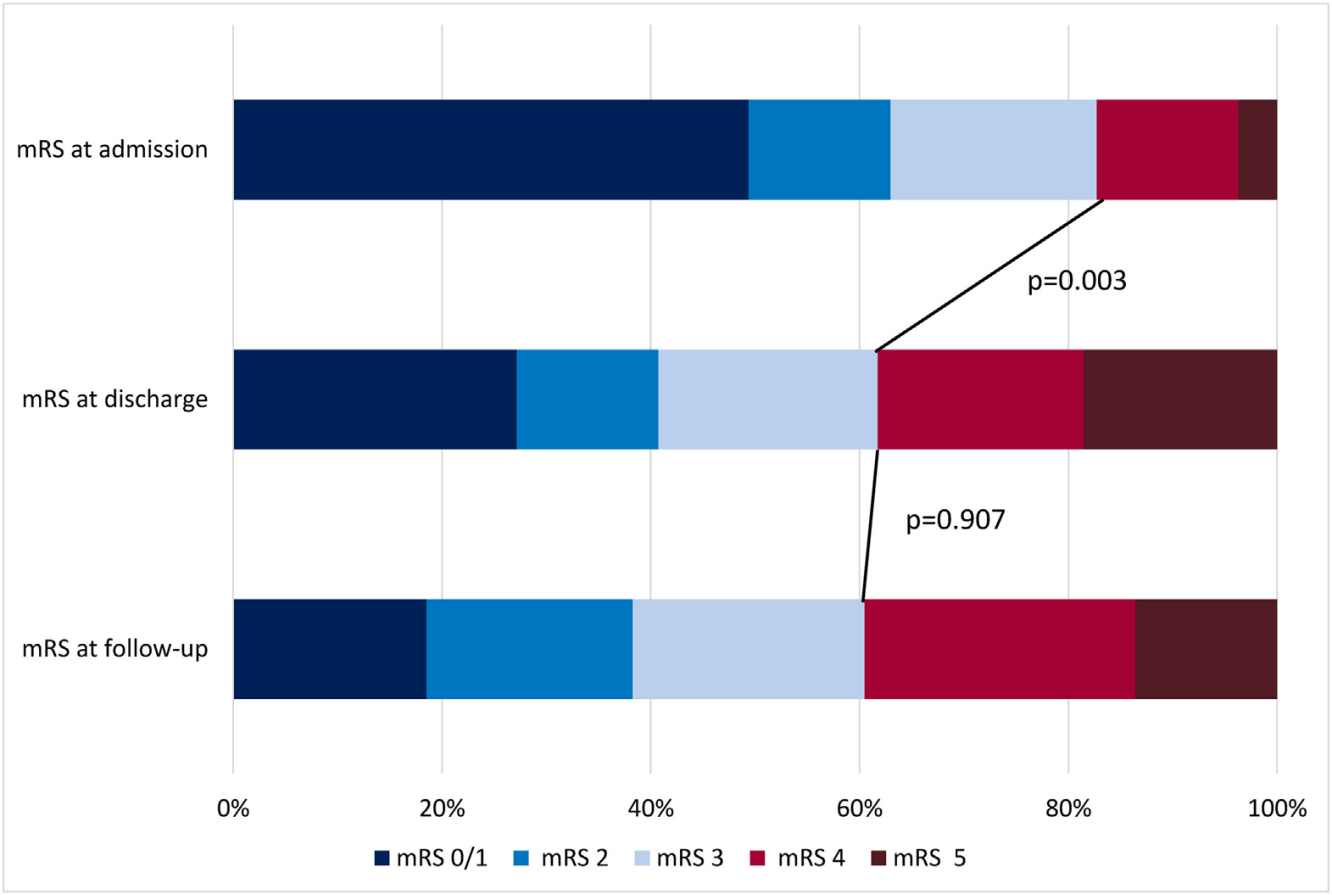
Modified Rankin Scale (mRS) at admission, discharge, and follow-up in patients with status epilepticus.

Before admission, a favorable mRS of 0–3 was present in 82.7% (*n* = 67), while an unfavorable mRS of 4–5 was seen in 17.3% (*n* = 14) of the SE population. On discharge, mRS decreased significantly with 31 patients (38.3%, *p* = 0.003) rated at an unfavorable mRS of 4–5. The mRS at follow-up did not differ from the one at discharge; 60.8% (45/74) presented an mRS of 0–3 and 39.2% (29/74) an mRS of 4–5 (Figure 1); seven patients did not report their mRS on follow-up. Regarding RSE and SRSE, a favorable mRS of 0–3 was present in 45.5% at discharge and increased to 70% at follow-up.

At discharge, the majority returned home (51.9%, *n* = 42), 32.1% (*n* = 26) entered a rehabilitation facility, 12.3% (*n* = 10) were transferred into a nursing home, and 3.7% (*n* = 3) to another hospital. At the time of follow-up, 21 patients (25.9%) lived at home without any help, 43 (54%) depended on aid of their families, and partners or of ambulatory nursing care, while 16 patients (19.8%) lived in a nursing home. A care level (Pflegestufe) was attributed to 56.8% and a grade of disability to 79% of the SE patients. Only 9 of 49 patients of working age (18.4%) had been employed at follow-up.

**Figure 2:**
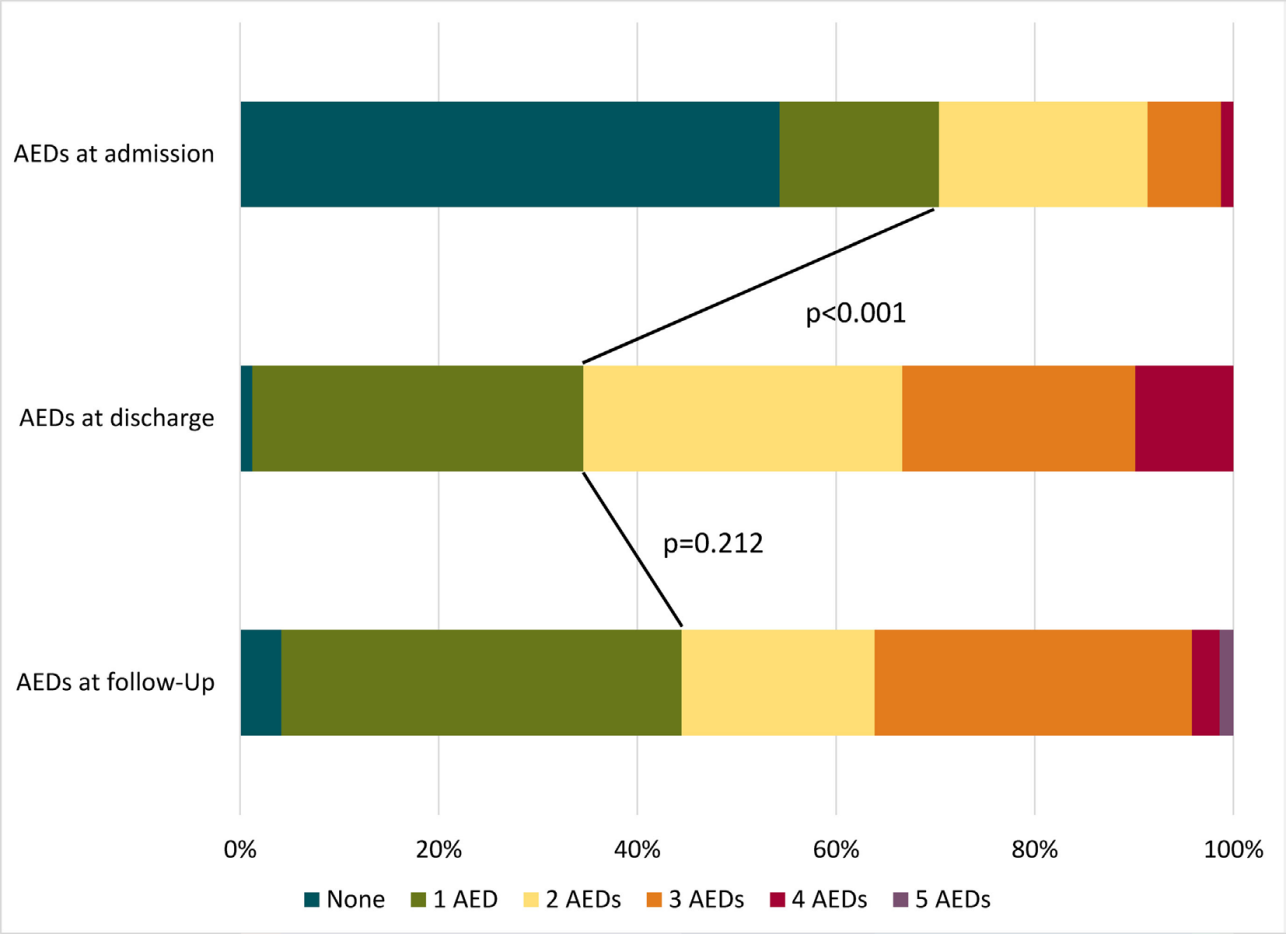
Number of used anticonvulsants at admission, discharge, and follow-up in patients with status epilepticus. AEDs, antiepileptic drugs.

Regarding healthcare resource utilization within the last 3 months, an ambulance transport to hospital was necessary in 13.2%. The mean number of outpatient consultations due to epilepsy amounted to 3.1 ± 3.3 (range 1–17). Overall, 22.5% (*n* = 18/81) of the SE patients were hospitalized due to epilepsy for a mean of 6.8 days within the last 3 months. Patients were in need of ancillary therapies, such as physiotherapy, occupational therapy, or speech therapy, with a mean number of 22.1 ± 18.3 (range 1–80) sessions within the previous 3 months. The number of AEDs did not differ at follow-up compared to discharge (see Figure 2).

The evaluation of QoL using the EQ-5D-Index showed 0.57 ± 0.36 on average (0 = death to 1 = full health). Most of the patients showed impairments due to side effects of anticonvulsants; the mean ABNAS score amounted to 34.9 ± 20.8 and 73.6% of the patients reported a high score (ABNAS 0–99, 99 = the worst score, >15=high score, and ≤15=low score) (23). Table 1 presents the socioeconomic characteristics and QoL in relation to the severity of SE. Admissions were more likely due to an acute symptomatic etiology in patients with RSE or SRSE (39.4%) than with a non-refractory course (16.7%, *p* = 0.021). Furthermore, the course of SE had a significant impact on the degree of disability, as measured by mRS at discharge [mRS 0–3 in 72.9% (35/48) vs 45.5% (15/33); *p* = 0.012]. Patients with RSE and SRSE were discharged with more AEDs [2.5 ± 1.1 (mean ± SD) vs 1.8 ± 0.9; *p* = 0.007], while this difference was not detectable at follow-up (*p* = 0.113). Patients with non-RSE were more frequently discharged home (64.6 vs 33.3%; *p* = 0.013), while more patients with RSE or SRSE were transferred into a rehabilitation center (54.5 vs 16.7%; *p* < 0.001). Concerning the QoL, patients after an RSE or SRSE achieved the same outcome as patients with a non-refractory course, as measured by NDDI-E, ABNAS, LAEP, QOLIE-31, or VAS.

### Comparison of QoL between Patients after SE and Patients with Drug Refractory Epilepsy and in Seizure Remission

For each patient after SE, one patient with DRE and one with SRE were matched by age and gender. The mean length of epilepsy amounted to 19.9 ± 17.6 years in DRE and to 17.0 ± 16.0 years in SRE. SE patients had a significantly shorter length of epilepsy (9.3 ± 14.2 years, *p* < 0.001 DRE and *p* = 0.001 SRE) at follow-up.

Table 2 shows the socioeconomic and clinical characteristics and QoL of SE patients at follow-up compared to patients with DRE and SRE. In the two control groups, significantly more patients lived at home independently than in the SE group (SE: 25.9%; DRE: 46.3%; *p* = 0.008, SRE: 64.6%; *p* < 0.001), in particular, in the cohort with SRE, no patient lived in a nursing home. Concerning marital status, 70.3% of patients with SRE were married in comparison to 55.6% (*p* = 0.048) of patients in the SE cohort. The mRS at follow-up documented a better state of health for both control groups (mRS 0–3: DRE 98.8%, SRE 97.5%) in comparison to the SE group (mRS 0–3 in 60.8%; *p* < 0.001 each). Regarding epilepsy-related healthcare resource utilization, about 22.2% (*n* = 18) of the SE patients were treated in a hospital, whereas fewer patients with drug refractory epilepsy (11.1%; *n* = 9, *p* = 0.057) and with SRE (1.2%; *n* = 1, *p* < 0.001) were hospitalized due to epilepsy within the last 3 months. Mean length of stay amounted to 6.8 ± 5.9 days (range 1–20) for SE, 7.0 ± 5.2 days (range 1–18) for DRE, and 7.0 days for SRE. An outpatient hospital treatment due to epilepsy was necessary for 14.3% patients with SE, 23.8% of patients with DRE, and 7.9% of patients in SRE. Patients in SRE were treated with a mean number of 1.3 ± 0.7 anticonvulsants (AEDs) and with significantly fewer AEDs than those with drug refractory epilepsy (1.9 ± 0.8; *p* < 0.001) or SE (1.9 ± 1.1; *p* < 0.001).

**Table 2:** Socioeconomic and clinical characteristics of patients with SE in comparison to matched epilepsy patients with drug refractory course and in seizure remission (*n* = 81 in each group).

	SE (*n* = 81)	Drug-resistant epilepsy (*n* = 81)	Epilepsy in SRE >1 year (*n* = 81)	*p* Value
Age (years) at survey				
Mean ± SD	58.7 ± 18.0	57.3 ± 15.6	56.2 ± 14.0	0.082 (SE vs DRE)
Range	21–97	21–94	20–87	0.001 (SE vs SRE)
Sex	% (*n*)	% (*n*)	% (*n*)	
Male	42.0 (34)	42.0 (34)	42.0 (34)	1.0
Female	58.0 (47)	58.0 (47)	58.0 (47)	
Marital status	% (*n*)	% (*n*)	% (*n*)	
Married or with partner	55.6 (45)	64.2 (52)	70.4 (57)	0.302 (SE vs DRE)
Divorced or in separation	6.2 (5)	7.4 (6)	9.9 (8)	0.048 (SE vs SRE)
Living with family/relatives	7.4 (6)	11.1 (9)	12.3 (10)	
Widowed	29.6 (24)	17.3 (14)	6.2 (5)	
n.a.	(1)		(1)	
Living situation	% (*n*)	% (*n*)	% (*n*)	
At home without help	25.9 (21)	45.7 (37)	62.9 (51)	0.008 (SE vs DRE)
At home with help (family, nursing service)	54.0 (43)	50.6 (41)	34.6 (28)	<0.001 (SE vs SRE)
Nursing home	19.8 (16)	2.5 (2)	–	
n.a.	(1)	(1)	2.5 (2)	
mRS at follow-up	% (*n*)	% (*n*)	% (*n*)	
mRS 0–3	60.8 (45)	98.8 (80)	97.5 (79)	<0.001 each
mRS 4–5	39.2 (29)	1.2 (1)	2.5 (2)	
Number of AEDs at follow-up	% (*n*)	% (*n*)	% (*n*)	
0	4.2 (3)	–	4.9 (4)	
1	40.3 (29)	40.7 (33)	65.4 (53)	
2	19.4 (14)	35.8 (29)	25.9 (21)	
≥3	36.1 (26)	23.5 (19)	3.7 (3)	
Number of AEDs (mean ± SD)	1.9 ± 1.1	1.9 ± 0.8	1.3 ± 0.7	0.632 (SE vs DRE); <0.001 (SE vs SRE)
Healthcare resource utilization within the last 3 months				
Inpatient treatment due to epilepsy	% (*n*)	% (*n*)	% (*n*)	
Yes	22.2 (18)	11.1 (9)	1.2 (1)	0.057 (SE vs DRE)
None	77.7 (63)	88.9 (72)	98.8 (80)	<0.001 (SE vs SRE)
Length of stay (mean ± SD)	6.8 ± 5.9	7.0 ± 5.2	7.0	
Outpatient hospital treatment due to epilepsy	% (*n*)	% (*n*)	% (*n*)	
Yes	14.3 (11)	23.8 (19)	7.9 (6)	0.105 (SE vs DRE)
None	85.7 (70)	76.3 (62)	92.1 (75)	0.2 (SE vs SRE)
NDDI-E	% (*n*)	% (*n*)	% (*n*)	
Major depression (>15 points)	32.8 (19)	36.8 (21)	20.3 (12)	0.79 (SE vs DRE)
No major depression (≤15 points)	67.2 (39)	63.2 (36)	79.7 (47)	0.128 (SE vs SRE)
LAEP	% (*n*)	% (*n*)	% (*n*)	
Total score (mean ± SD)	41.9 ± 10.4	40.5 ± 11.8	37.0 ± 11.8	0.375 (SE vs DRE)
Range	14–63	19–69	19–45	0.011 (SE vs SRE)
QOLIE-31 (1–100, 100 best QoL)	% (*n*)	% (*n*)	% (*n*)	
Overall T	43.5 ± 13.7	44.8 ± 12.8	51.8 ± 12.5	0.698 (SE vs DRE); <0.001 (SE vs SRE)
Quality of Life T	41.7 ± 11.5	46.6 ± 9.7	50.2 ± 11.2	0.013 (SE vs DRE); <0.001 (SE vs SRE)
Seizure Worry T	51.3 ± 12.3	48.9 ± 10.5	56.0 ± 9.7	0.084 (SE vs DRE); 0.042 (SE vs SRE)
Emotional Well-Being T	43.8 ± 11.2	46.3 ± 10.7	49.4 ± 10.8	0.296 (SE vs DRE); 0.009 (SE vs SRE)
Energy-Fatigue T	43.6 ± 9.2	46.3 ± 9.8	49.6 ± 10.1	0.114 (SE vs DRE); 0.002 (SE vs SRE)
Cognitive Functioning T	45.8 ± 12.6	46.8 ± 12.0	50.6 ± 11.9	0.832 (SE vs DRE); 0.011 (SE vs SRE)
Medication Effects T	49.0 ± 10.7	50.8 ± 9.6	53.8 ± 9.3	0.328 (SE vs DRE); 0.011 (SE vs SRE)
Social Functioning T	44.3 ± 11.8	45.2 ± 10.5	52.0 ± 10.4	0.909 (SE vs DRE); <0.001 (SE vs SRE)
VAS (mean ± SD)	48.3 ± 24.5	55.3 ± 22.9	65.3 ± 22.1	0.269 (SE vs DRE)
				<0.001 (SE vs SRE)

*AEDs, antiepileptic drugs; SE, status epilepticus; DRE, drug refractory epilepsy; mRS, modified Rankin Scale; NIDDI-E, Neurological Disorders Depression Inventory for Epilepsy; LAEP, Liverpool Adverse Events Profile; QOLIE, Quality of Life in Epilepsy Inventory; VAS, visual analog scale; SRE, seizure remission*.

A major depression, as indicated by NDDI-E, was found in nearly one-third of patients with SE (32.8%) and DRE (36.8%), but only in 20.3% of patients with SRE. Side effects intensity evaluated by the LAEP score was 41.9 ± 10.4 in SE patients, 40.5 ± 11.8 in DRE patients, and lower in SRE patients (37.0 ± 11.8; *p* = 0.011). A better QoL, measured by QOLIE-31 and VAS, was seen for patients in SRE in all categories compared to patients after SE. Regarding the subcategory QoL, both patients with DRE (*p* = 0.013) and SRE (*p* < 0.001) scored better than patients after SE; for details, please refer to Table 2.

## Discussion

This study on QoL and socioeconomic outcome after an episode of SE is the first comprehensive evaluation to address sequelae and outcome of adult patients with SE and compare them with matched epilepsy controls with a drug refractory course or in SRE. We can show that patients with RSE and SRSE had a deterioration in neurological functions at discharge, which can be set off at follow-up. Furthermore, patients after RSE and SRSE may achieve an equivalent QoL compared to patients after a non-RSE. Despite persistent and increased neurological deficits in patients after SE, these patients may achieve similar QoL values to patients with DRE who have no neurological deficits. However, QoL was reduced in all subcategories of QOLIE-31 when compared to patients in SRE.

Our findings underline the need for an efficient therapy of RSE and SRSE, as these patients will, on average, achieve outcomes comparable to patients with a non-RSE and patients with DRE. That is remarkable as these patients are suffering from persisting neurological deficits, as measured by mRS, and show an increased need for assistance in daily activities. Furthermore, patients with RSE and SRSE improve in neurological outcome over time. This is in line with previous findings, as reported by Lai et al. (29). They reported functional outcome using the mRS in patients with prolonged RSE on admission, discharge, and 1 year after discharge. In their cohort, a favorable outcome of mRS 0–3 increased from 11.5% of patients at discharge to 17.1% of patients at follow-up (29). Ferlisi and Shorvon reported on long-term outcomes of RSE and SRSE, providing mortality rates and data on neurologic defects in 596 cases (4). Overall, 35% of the patients were able to return to baseline, while 13% suffered a severe neurologic deficit, a further 13% had a mild neurologic deficit and 4% had an undefined neurologic deficit. Mortality accumulated to 35% (4). The preliminary report of the global audit from 44 countries on treatment of RSE and SRSE (30) showed a favorable outcome with an mRS of 0–3 in 36% of the patients at discharge (26.6% = mRS 0–2; 9.4% = mRS 3), which improved to 63.8% at follow-up (42.6% = mRS 0–2; 21.3% = mRS 3). The follow-up was available for 108 patients with an obvious selection bias, as noted by the authors.

A retrospective study from India used the Glasgow Outcome Scale (GOS) to describe outcome. They reported that SRSE had a worse outcome after 6 months in comparison with RSE (33.3 vs 57.1%; *p* = 0.055) and non-RSE (33.3 vs 79.1%; *p* < 0.0001) (31). These findings are well explained by encephalitis as the main underlying etiology in SRSE cases reported from India, an etiology that is independently associated with a poor outcome.

Sutter et al. reported on identification of predictors for outcome involving clinical features such as age, history of prior seizures or epilepsy, SE etiology, level of consciousness, and seizure type at SE onset (6). Determination of predictors from our study is hindered by the limited sample size. However, overall mortality rates of the overall SE group and outcome measures (mRS or GOS) are in line with previous publications (4, 5, 29, 31).

Patients after SE achieved QoL scores comparable to patients with DRE who had never suffered from SE. Given the neurological deficits in the SE group, these seem rather surprising. Strong determinants of reduced QoL in DRE are depression and anxiety, as shown by multiple studies (9, 32, 33). Furthermore, tolerability and efficacy of AEDs, employment, seizure frequency and semiology, and comorbidities will influence some aspects of QoL in DRE (9, 32–35). These factors are also present in patients after SE, e.g., depression in one-third of our SE cohort, and should be kept in mind during rehabilitation and further outpatient treatment. Use of inpatient and outpatient services after SE remains high, showing the ongoing need for neurological care to this potentially vulnerable patient group. Most of the studies on outcome of SE focused on the first months to a year after discharge, which might influence the QoL outcome. DRE patients in our study suffered for two decades from epilepsy, which likely explains some of the deterioration in QoL.

### Limitations of the Study

Despite a careful design and strong efforts to gather follow-up data, this study has certain limitations. Direct comparison to other studies is difficult because of different healthcare settings, differences in etiology between different regions, age considerations (children are not included in our study), and varying treatment approaches. Definition of RSE might differ among studies with varying amount of drugs or time passed to define an SE as being refractory.

Due to the study design, which implies a questionnaire that was filled out by patients or their families, we cannot exclude a misunderstanding sometimes leading to incorrect answers. Furthermore, results of this survey are probably biased by selection due to the SE-associated mortality (overall 18.8%) and morbidity that were also described in other studies (30). Morbidity and mortality after discharge might explain the low responder rate of 15%, and mortality at discharge and during follow-up might be the main confounder in our study. The average in-hospital mortality in our SE cohort is 5.8% for non-RSE and 20.1% for RSE and SRSE (1) and matches nationwide evaluations of mortality in SE (3). We have to assume that participating patients were able to write or communicate with their relatives to complete the questionnaire. Adults who depend on help of their family members, who live in a nursing home, or who suffer a severe disability might be underrepresented in this evaluation. As we used patient questionnaires to collect data regarding resource utilization, the possibility of incomplete patient recall in some of the categories cannot be excluded and could have resulted in an underestimation of resource use. Another limitation of the study is the relatively short evaluation period of 3 months, which could have led to large variability in estimates.

## Conclusion

Patients after SE show substantial impairments in their QoL and daily life activities. However, QoL is comparable to patients with DRE, despite more SE patients being affected by neurological deficits. Further studies and treatment evaluations are warranted to answer questions on the outcome of SE patients in the future, especially if new treatment strategies might improve initial outcome and reduce in-hospital mortality. In the long term, patients with RSE and SRSE might have a favorable outcome regarding QoL and neurological functions compared to patients with a non-refractory course. This underlines the need for efficient therapeutic options in these challenging situations.

## Ethics Statement

We confirm that we have read the Frontiers in Neurology position on ethics and procedures and confirm that this report is consistent with these guidelines.

## Author Contributions

LMK and AS generated the research idea, study design, and concept. LMK, FP, and AS acquired the data. LMK and AS analyzed the data and drafted the work. LMK, SK, FP, FR, and AS made critical revisions for important intellectual content and interpreted the data. LMK and AS wrote the manuscript. LMK, SK, FP, FR, and AS approved the final manuscript.

## Conflict of Interest Statement

LMK reports personal fees from Desitin Arzneimittel. SK reports personal fees from Desitin Arzneimittel and UCB Pharma. FP reports industry-funded travel with support of Desitin Arzneimittel, Eisai, and UCB Pharma obtained honoraria for speaking engagements from Desitin Arzneimittel, Eisai, and UCB Pharma, was part of a speaker’s bureau of Desitin Arzneimittel, Eisai, and UCB Pharma. FR reports grants and personal fees from Eisai, UCB Pharma, Desitin Arzneimittel, Novartis, Medtronic, Cerbomed, Sandoz, Shire, and the VfA (Verband der Forschenden Arzneimittelhersteller) and grants from European Union and Deutsche Forschungsgemeinschaft. AS reports grants and personal fees from Bayer HealthCare, Boehringer Ingelheim, Desitin Arzneimittel, Eisai, LivaNova, Pfizer, Sage Therapeutics, UCB Pharma, and Zogenix. The reviewer FW and handling editor declared their shared affiliation.
